# Insanity as a Symptom of Bright’s Disease1Address in Mental Disorders delivered before the Medical Society of the State of Pennsylvania, at Pittsburgh, June 10, 1890, from advance sheets from the Transactions.

**Published:** 1890-10

**Authors:** Alice Bennett

**Affiliations:** Norristown, Pa.


Buffalo Medical and Surgical Journal

VOL. XXX. OCTOBER, 1890. NO. 3.

### Original Communications.

### INSANITY AS A SYMPTOM OF BRIGHT'S DISEASE.[1]

BY ALICE BENNETT, M. D., PH. D., of Norristown, Pa.

I HAVE wished, if possible, to make this half hour, during which I have the honor to address you, of some interest, more especially to the general practitioners of medicine, who make up the great bulk of the society. To that end I have determined to ask your attention, not to a review of the year's work in the department which I represent, a custom which has come to be "honored in the breach" quite as often as in the observance, but to a field where the labors of the specialist and of the general practitioner meet in close relation, and where the observations upon either hand have been few and incomplete. My subject is Insanity as a Symptom of the so-called Bright's Disease, or Diseases, a name which, unsatisfactory as it has become, we are yet not prepared to throw away.

The growth of our knowledge has hitherto been hindered by the too sharply drawn line which has divided the doctors who study the diseases of the body, on the one hand, from those who are studying the diseases of the mind, upon the other. The general practitioner has been too apt to stand aloof and gaze upon the spectacle of the "human mind diseased" as a something outside his ken, under the influence of forces new and strange — as "outstanding exceptions of humanity, unintelligible except on psychological hypotheses"— to which he supposes the alienist to possess some key all his own; while, on the other hand, the "insane doctors" have been so much absorbed in following and classifying the bewildering windings of the disordered mind that they have too often forgotton the body.

But I take it as a cheering sign when, from the ranks of the alienists themselves, come words like these[2] :

1. Address in Mental Disorders delivered before the Medical Society of the State of Pennsylvania, at Pittsburgh, June 10, 1890, from advance sheets from the Transactions.
2. John Battey Tuke, *Nineteenth Century*, June, 1889.

The psychological theory of insanity has prevented advance in the study of those forms of disease of which mental alienation is the most prominent, but by no means the sole, or even the most important, symptom. Blinded by this glamour of psychology, we have lost nearly a century of observation, and have frittered away the lives of hundreds of good men. Let me repeat here also the words of the member who last occupied this chair[1] : If there be one thought in this connection which ought never to be forgotten, it is, that *human mind diseased is the human mind still.* The coming on of insanity marks *not* the appearance of a *new* entity or a *new* force, *Our so-called mental diseases are simply groups of symptoms.*

This is the fundamental thought which I want to emphasize. Insanity is a symptom, or group of symptoms ; not always, not in the majority of cases, at least in its beginnings, a symptom of integral disease of the organ whose perverted action it is, but of faulty conditions external to it. Whatever the initial step leading to disordered brain action, (I am speaking now of "ordinary insanity" in the sense used by Sankey, excluding that which is organic or developmental,) the remote effects are similar ; whether beginning as mania or melancholia, it ends sooner or later (*i. e.*, if the morbid process be not checked,) in dementia, that is, the limited function of a more or less impaired brain tissue. To the general practitioner alone, in the great majority of cases, comes the opportunity to study insanity in its inception, and to investigate the perverted bodily functions which determine the nutritive changes leaning to disordered brain action, when only preventive measures may often be attended with gratifying results.

When we consider the marvelously delicate structure of the brain, whose susceptibility to every impression is, in fact, its function, is it not self-evident that any defect in the nutritive processes upon which its integrity depends, any deterioration in the quality of the blood or in the manner of its supply, will be liable to influence its action ? And this, by a majority of the writers on mental diseases, is conceded in theory, if denied in practice.

*Schroeder von der Kolk* says[2] : "Among the causative forces acting on the brain, the first place must be given to the blood." Blandford says[3] : "Could we comprehend the blood supply of the brain beyond all manner of dispute, we should go far toward explaining most of the phenomena of brain function and of brain

1. Dr. H. C. Wood, 1888.
2. The Pathology and Therapeutics of Mental Diseases.
3. Insanity and its Treatment, Edinburgh, 1884.

disorder." And again: "The life and functions of the highest cerebral centres are disordered by interruption in their natural nutrition; if there is a defect—an impoverished blood, or a blood poisoned by deleterious ingredients, the effects must be visible in the functions of the brain."

This is logic and common sense; it is also physiology, and it is difficult to follow this author when, in his chapter on pathology, he reviews the organs most concerned in a pure and equable blood supply, as follows: "*Hearts* are frequently morbid, but we are not to connect these with the outbreak of insanity." "In the pathology of commencing insanity the *kidneys* play a very unimportant part." "I believe the *liver* has little to do with the pathological condition of a patient who has recently become insane," etc. Griesinger says: "Many cases that terminate fatally in the acute stage present pretty much the appearance of the normal brain; often enough to lead to the conclusion that the symptoms during life were due to some disturbance in the process of nutrition as yet unknown to science." Sankey[1] separates what he calls "ordinary insanity" from paresis, epilepsy, organic and developmental insanity. Speaking of the etiology of "ordinary insanity," he says: "The disease consists in a morbid state of the blood, or of the processes concerned in nutrition."

His *résumé* of the morbid anatomy in insanity is at once so comprehensive and so concise, and bears so directly upon our subject, that I make no apology for quoting it:

> During the earlier period of the disease (Sankey regards mania and melancholia as only differing manifestations of one morbid process), the symptoms are due to an alteration in the blood, in its quality and in its amount; there is some congestion with interstitial deposit of serum and of protein compounds; then atrophy of the brain substance and hypertrophy of the vessels. At first the symptoms are due to the circulation of impure blood; they next are due to excessive supply; then they evidence the imperfect function of an altered cerebral tissue.

Coulston says of melancholia: "It is a constitutional disorder of the brain developed out of hereditary tendency and excited into action by peripheral disease in some other part of the body." Accepting, then, the self-evident fact which, moreover, has the support of the high authorities named, that "ordinary insanity" in its first stage depends upon some defect in the quality, or the mode of supply, or both, of the blood, we can hope to get some

1. Lectures on Mental Disease, London, 1884.

light upon the causes of insanity only by studying the organs which have to do with the blood production, purification and supply. In this vast field, where so many avenues of inquiry open, I shall attempt no more than to make some suggestions along one path in which my own clinical experience has happened to lead me.

It is understood, of course, that no organ, or system of organs, acts independently; that there can be no absolute separation of the study of one from the study of another,—as, for example, the liver, kidneys and heart, often related links in one morbid process—nevertheless each has its definite function, interference with which is followed by equally definite consequences.

By "Bright's disease" we no longer understand a morbid process affecting the kidneys only, but whether we consent to accept the kidney lesion as a part of a general "arterio-capillary-fibrosis" (after Gull and Sutton), or with Dr. A. V. Meigs, look upon it as a localized expression of a general "endarteritis," the fact is undisputed that at some stage in the train of morbid processes, covered by the monumental name "Bright's disease," lesions of the kidney do exist; their function is interfered with; to a greater or less extent they fail to separate certain waste products from the blood, which being retained and circulated through the body produce toxic effects which we have been accustomed to group under the general term "uremia," or "uremic poisoning."

I must pass by, as not essential to my purpose, any consideration of the various theories concerning the nature of these nitrogenous waste products, and confine myself to some observations upon their effects, which my experience has led me to believe are more varied and far-reaching than has generally been supposed.

There has been a general impression abroad that diseases of the kidney are not common among the insane, founded upon statements to that effect in most of the text-books and perpetuated by the very general absence of systematic and careful observations in this direction. Griesinger, in his work on Insanity, says: "Anomalies in the urinary secretion may be much more frequent," *i. e.*, among the insane, "than is generally supposed. Unfortunately, any reliable researches upon this important subject are still wanting."

My attention was first arrested by the clinical observation of the very constant coincidence of some form of mental pain or distress, *i. e.*, melancholia, with the physical signs in the urine of disturbed kidney action; this is not invariable, but the rule. We have cases of undoubted mania associated with a uremic condition, and, on the other hand, cases of melancholia without it; as, for example, in some conditions of grave heart lesions with general debility, and some transitory cases associated with disturbed liver action with the uric-acid formation.

[A. Haig, in an article in the *London Practitioner*, Vol. xli., No. 5, on "Mental Depression and the Excretion of Uric Acid," speaks of the demoralizing influence of uric acid upon the nerve centres, and explains the well-known fact, that states of mental depression are intensified in the morning by the increased alkalinity of the blood at that time, and consequent greater solubility of uric acid. We know that our melancholic patients are worse and that suicidal impulses are to be specially guarded against in the early morning.]

Briefly formulated, my experience has led me to believe—

1. That, contrary to generally received opinion, affections of the kidney are very common among the insane.

2. That "uremic poisoning" is one of the most frequent causes of insanity.

3. That while the mental manifestations may be as varied as there are different centres subjected to irritation by these unknown poisons, the most prominent and constant symptom is some form of *mental pain*, which may range from simple depression, through all degrees and varieties of delusions of persecution, self-condemnation and apprehension, with or without hallucinations, up to a condition characterized by a frenzy of fear, with extraordinary motor excitement, and rapid physical prostration, the "grave-delirium" or "typho-mania" of some authors.

4. That the motor centres are specially liable to affection, as evidenced by the restlessness and incessant activity of many cases, less frequently by convulsions and convulsive twitchings; occasionally by choreic movements; occasionally by cataleptoidal states.

Undoubtedly there is much more of Bright's disease" in the community than appears on any record book, the interstitial form often running a very long course, frequently unrecognized. Persons subject to "bilious attacks" and "sick-headaches"; to obscure neuralgias; to crawling sensations (often described to me "like the flowing of water" over the part affected) in the head and especially in the back of the neck; people who are "tired all the time," who have sleepless nights, or occasional night terrors; who have unexplained attacks of sudden weakness, or periods of low spirits without cause; who show an unnatural irritability, or a gradual change of character or disposition; those who are subject to gout, rheumatism, chorea, skin eruptions, to itchings of the surface of the body, either local or general—all these may well be suspected of dangerous tendencies.

I need not say that numberless cases of slow kidney trouble live long and fairly comfortable lives without showing any mental disturbance, and that many others run a rapid course to death without such complication. We must assume, in some cases, a toxic impression of overwhelming power, but, doubtless, some brains are predisposed, by inheritance or otherwise, to an easy overthrow of the mental balance. This seemed plain in many of my cases. In such a one, given a chronic nephritis, or even without it, insanity may be induced by anything that increases the burden thrown upon the kidney, diminishes its working-force, or interferes with other excretions. Such causes are: improper diet; long-continued constipation ; sudden exposure to cold ; pregnancy, or any unusual interference with the circulation ; overwork of body or mind and especially worry ; intercurrent disease, or anything that depresses the system and lessens its power of resistance. The influenza epidemic in the beginning of the present year sent us a number of cases of melancholia which belong in this category. (See Cases LVI., LVII. and LIX.) A factor which cannot be left out of account in these cases is the heart; whether a coincident or resultant change, we know that, with diseased kidneys, we are apt to have abnormal hearts, and it is an interesting question to what degree mental disturbance may be aided by some modification in the supply of blood to the brain, due to normal heart action.[1]

Dr. Landon Carter Gray, of New York, read a very interesting and suggestive paper before the American Neurological Association, in 1889, on "Three Diagnostic Signs of Melancholia," with notes of sixteen cases illustrating the *association of mental depression, insomnia, and post-cervical ache*, which he has found so constant in his practice. Dr. Gray says: "The simple forms of melancholia are often extremely difficult to diagnosticate, especially in the early stage, as the reasoning powers, the memory, and the perceptions are then often seemingly unimpaired or not more affected than is possible from a myriad unimportant causes. Patients suffering from this mental disease too frequently figure as neurasthenics to be confidently treated as such, until some determined and frightful suicidal or homicido-suicidal attempt throws startling light upon the true nature of the malady. These, too, are the cases of unaccountable suicide which puzzle friends, and competing newspaper

1. In the Address in Mental Disorders, for 1884, the writer gave an analysis of 500 cases of insanity, 20 per cent. of which had some heart affection.

reporters account for so satifactorily and sensationally upon some theory of rejected love or high-flown sentimentalism. Any certain diagnostic symptoms in this class of cases should be, for these reasons, of value. . . . So firmly have I come to rely upon the association of this symptomatic triad," [*i. e.*, mental depression, insomnia, and post-cervical ache,] "that I have lately made a diagnosis in two cases by means of it. The first patient was a gentleman who came to me complaining of a distress in the back of the head and neck, which at times was painful. I learned from him that the onset dated back to six years ago, when, as he said, he had been run down and depressed. I then told him that I would outline to him his symptoms at that time, and I proceeded to tell him that he had been very much depressed, had not been able to sleep, had thought of committing suicide, had been slightly confused in mind, and had remained in this condition for several months. He was amazed, and asked me if I was a mind-reader, finally admitting that he had passed through just such an attack of melancholia, which he had concealed from everybody, because he was then living in Burmah in the employ of the English Government, and was afraid that he would lose his position if thought insane."

I fully concur in all that Dr. Gray has so well said in this paper; but, in going a step farther and investigating the *causes* of insomnia and post-cervical ache, both among the most common symptoms of "uremic" blood poisoning, we shall find additional aids to diagnosis and safeguards against catastrophes like those mentioned.

In cases of Bright's disease with sudden invasion of melancholia, there is one feature so constant that I have come to regard it as diagnostic: it is the *sense of impending danger*, the overwhelming *fear* of some threatening calamity, which inspires the one irresistible impulse to "get away" which dominates the individual for the time, and under the influence of which he often jumps out of the nearest window. (See Cases LV., LVIII., XLVII., and others.)

To prevent insanity by recognition and treatment of the conditions leading to it will be our aim; frequently, however, so insidious is Bright's disease, and so unwilling are people generally to appear to make much of their little ailments, which would be such valuable indicators if revealed, that we know nothing of the state of affairs until some catastrophe has taken place. Even then it is worth much to be able to say why it has occurred, and even in unpromising cases gratifying results sometimes follow prompt treatment in the lines indicated, but prognosis must always be guarded.

I think that I can serve you best by presenting some clinical notes of cases with comments; but before going on to speak of my own experience, I will ask you to look at the literature of the subject.

Books on diseases of the kidney say almost nothing of the effects of retained nitrogenous waste products upon the nervous system, except convulsions and coma, generally preceding death.

From all the works of insanity accessible to me I have gathered everything bearing on this subject that I could find.

From Bucknill and Tuke:[1] "The kidneys are remarkably free from disease in all forms of insanity. We have met with three cases of Bright's disease among the insane, and we have found the experience of others of a similar nature." They quote, with evident surprise, Howden, who had admitted twelve cases of albuminuria in three years, and who, in 235 post-mortem examinations, had found kidney disease in 86.

They admit two genuine cases of "insanity co-existent with the waxy form of Bright's disease," mentioned by Dr. Wright, in the *Report of the Royal Edinburgh Asylum*, for 1871, and speak also of a similar case recorded by Dr. Wilkes in the *Journal of Mental Science*, for 1869.

In connection with gout, Bucknill and Tuke say:

> Outside the walls of asylums cases are frequently met with which are marked by *unfounded dread*, especially on awaking in the morning, in which there is a gouty diathesis, and suspicion is aroused that there is a causal relation between the bodily condition and the mental anguish. This suspicion is confirmed by the marked success of treatment founded upon this supposition.

Schroeder v. der Kolk, under "Sympathetic Insanity," records two cases of insanity accompanying vesical catarrh, of which one recovered; in the other an affection of the kidneys supervened, and the patient died.

Sankey found adhesion of the capsule of the kidney in one-half his cases post-mortem, and, "in a large number, other evidences of disease, as atrophy of the cortex, fatty degeneration, waxy disease, etc." This author does not seem to have made any study of the kidneys during life. The presence of kidney changes post-mortem he regards as "evidence that the blood has been impure" (emphasized by him as the first step in the production of "ordinary insanity," as quoted above); nevertheless, he concludes by saying: "I do not consider the true pathology of insanity to have any necessary relation to kidney disease."

1. Psychological Medicine.

Gowers calls attention to organic changes (as apoplexy), which may ensue from atheroma of the arteries in advanced Bright's disease.

Griesinger[1] says: "Bright's disease to which any etiological relation to insanity could be attributed is very rare indeed." One would much like the complete clinical record of a case like his No. 12, of which the following is a condensed abstract: "Delirium occurring in second pregnancy; formication and smarting over whole surface; a general sense of ill-being; sleeplessness; ringing in ears; vertigo; pulse hard; slight cardiac hypertrophy; recovered, and relapsed in the following year."

Or his No. 8: "Man; hepatitis in the beginning; variable temper; pain in lumbar region; burning in urethra and bladder; at times had gravel; died in a few months."

Blandford speaks of a variety of melancholia "usually ascribed to dyspepsia or disorder of the stomach and liver." He has also noticed that "melancholia often comes on, as a precursor of death at the close of other diseases."

In Clouston's[2] very interesting work I find more bearing upon this subject than in any other I have read, although I cannot always agree with this author in his interpretation of facts. For example, his two cases of diabetic insanity, which have been extensively quoted in other text-books. In case No. 2, a man who died after melancholia of two year's duration, with delusions of persecution, the diagnosis rested entirely upon one examination of the urine made near the close of life; no symptom had led to the suspicion of diabetes, and there was no post-mortem examination.

That some amount of sugar in the urine is frequently associated with chronic Bright's disease is well known; and I have twice met the condition noted in my Case VI., where, shortly before death sugar appeared in large quantities in the urine, from which it had previously been absent.

His Case No. 1 was a "woman, aged fifty-nine; agitated melan, cholia; toward the end, sleepy all the time; urine never very copious; ordinary treatment of diabetes of no avail."

Clouston recognizes a "variety of mental derangement, half delirium and half mania, which results from uremic poisoning, which he names the "insanity of Bright's disease." . . . .
"Usually occurring in chronic cases with contracted kidneys, where

1. Mental Pathology and Therapeutics.
2. Clinical Lectures on Mental Diseases, London, 1883.

there has been enlargement of the heart and a tendency to dropsy for some time," and he gives one illustrative case.

He has also noticed in such cases "mania of a delirious character, with extreme restlessness, with remissions, attended with great prostration" (the equivalent of the "grave delirium" of some authors, of which I shall want to speak again).

I was especially interested in Dr. Clouston's studies of melancholia, which is the one form of insanity I so constantly find associated with defective kidneys. He considers it under eight heads, as follows:

1. Simple melancholia.
2. Hypochondriacal melancholia.
3. Delusional melancholia.
4. Excited or motor melancholia.
5. Resistive melancholia.
6. Epileptiform melancholia.
7. Organic melancholia.
8. Suicidal and homicidal melancholia.

These divisions are convenient, but it is needless to say that (excluding "organic," which seems to me out of place here) they are descriptive of mental states, rather than varieties of disease; often transitory, passing one into the other in the same case. Etiologically, I would consider them all as one, including also stuporous melancholia, which Clouston considers under the separate general head "stupor." Of the "motor" and "epileptiform" varieties, he remarks that they are "specially liable to skin irritations, itchings, boils," etc.

The treatment that this author has found of most value in melancholia is, to me, significant: milk is his "sheet-anchor;" farinaceous and fatty foods; an abundance of fresh air; baths, and especially Turkish baths, of which he says: "I have seen many chronic, incurable cases of melancholia improved by a course of Turkish baths."

In our own country there have been some special contributions:

In 203 autopsies at the Government Hospital for the Insane, at Washington, D. C., about one-sixth of the kidneys "presented alterations sufficient to constitute disease."

Prof. William Osler reported to the Philadelphia Neurological Society, in 1888, three cases of insanity associated with "Bright's disease":

Case I. — Man, aged forty-two, known to be the subject of "Bright's disease;" after three or four days of violent mania, died in a comatose condition.

Case II. — Man, the subject of interstitial nephritis, admitted to the University Hospital in a semi-stuporous condition; had previously been maniacal, and had delusions of persecution; committed suicide by jumping from an upper window.

Case III. — A man, with chronic "Bright's disease," who for a time refused food under the influence of delusions of persecutions; afterward improved.

Dr. L. Bremer, of St. Louis, in a paper read before the State Medical Society of Missouri, in April, 1888, details seven cases that have come under his observation:

Case I. — Unmarried woman, thirty-eight; rheumatism and chorea at fourteen; albuminuria, following fall into cold water, of four years' duration; a second exposure followed by scanty secretion of urine, insomnia, irritability, mania of two days' duration, followed by melancholia with self-condemnatory delusions; attempted suicide on the ninth day, resulting in excessive hemorrhage, followed by sleep and rapid recovery. The urine contained albumin, hyaline and epithelial casts, pus, and blood corpuscles, disappearing with complete restoration to health.

Case II. — Man, a drinker; acute rheumatism, with fever, tremors, spastic state of muscles, delirious insanity marked by intense restlessness and vivid hallucinations; urine contained enormous amount of albumin, hyaline and blood casts, which almost disappeared during a remission of three weeks, when mind was clear, and again increased with a relapse which ended in coma and death eight weeks from the inception of the disease. No post-mortem examination.

Case III. — Woman, forty-eight; injury to head in childhood; puerperal mania at thirty-four; six years later, rheumatism with intense insomnia preceded a melancholia of several months' duration. Last attack followed prolonged exposure in a railroad accident; active delirium, with menacing hallucinations, coma; death one month from beginning of the attack. Albumin, epithelial and hyaline casts in urine.

Case IV. — Woman, fifty-five; sciatica for ten years; an exceptionally severe attack, followed by mental confusion, quickly passing into coma and death. Urine contained epithelial and blood casts, and pus corpuscles.

Dr. Bremer's Cases V., VI., and VII. are somewhat complicated, and I pass over them here.

Dr. E. A. Christian, of East Michigan Asylum, reports[1] thirty-

1. Journal of American Medical Association, March, 1889.

seven out of 2,600 cases of insanity admitted to that hospital, "in which the appearance of grave disturbances of nutrition had been coincident with albumin and tube-casts in the urine; in only about a dozen could it be said that the mental manifestations were not dependent upon, or modified to some extent by, the renal disease." Of these cases he makes two divisions: One, the "uro-toxic;" two, the "vascular." I give condensed abstracts of his five cases, illustrating the first class, which he considers much less common than the second. (In this my experience is widely at variance with Dr. Christian's.)

CASE I.—Woman, thirty-four; convulsions, followed by insanity, at a miscarriage three years before. A second attack, also puerperal, two years later. Third and last attack also puerperal; "grave dyspeptic disorders and failing vision" in interim. Mostly "confused and restless;" a low muttering delirium. Died semi-comatose, in the third month of attack. Urine contained albumin, waxy casts, and debris. No autopsy.

CASE II.—A year of dyspeptic troubles, headache, frequent vomiting, eczema, etc., was followed, successively, by delusions of suspicion, hallucinations of sight and hearing, low delirium, and, near the close, intense excitability of the motor-centres, with spasms of all the voluntary muscles. Albumin, hyaline and granular casts in the urine. Death in four months. No autopsy.

CASE III.—Male, eighty-one; restless and irritable for eighteen months. Extravagant ideas, followed by delusions of fear, merging into general mental confusion; coma of five days, death. Albumin and casts in the urine.

CASE IV.—Male, forty-five; sequence of symptoms as follows: Peculiar and forgetful; confused; hypochondriacal; suspicious; religious delusions. Died, semi-comatose, in four months. No autopsy. Albumin and casts in urine.

CASE V.—Woman, fifty-seven; a fever of some sort, followed by causeless worrying, profound depression, delusions of suspicion, edema; albumin and casts in urine; asthenic; paroxysmal, asthmatic seizures, with cough; died of pleuritic effusion, more than two years from the beginning of insanity.

While I do not agree with the last-named writer as to the preponderance of the "vascular" over the "uro-toxic" cases, I freely concede the frequency and importance of the former class, among which belong many of our early apoplexies. I want to say also, in passing, that the relation between paresis and "Bright's disease" seems to me to require further investigation; we know that many cases of paresis are associated with kidney disease, and I have seen cases, beginning as melancholia with uremia, pass into a condition similar to, if not identical with, paresis.

The cases which I present are all taken from the records of the Department for Women of the Norristown Hospital for the Insane. They are divided into clinical groups, and are condensed as much as possible.

*First group.*— Cases (12) rapidly fatal (twelve days to three months). I am persuaded that to this class belong many of the cases variously designated as "typho-mania," "grave delirium," etc., by different writers. Nothing more distressing, nothing more hopeless of even amelioration, can come either to the general practitioner or the specialist. Their characteristic features are intense motor excitement, with rapid physical prostration, a condition graphically described by Spitzka as follows:

> Insomnia and inability to think, increased irritability, and a sense of impending misfortune precede the outbreak, which is often so sudden as to suggest the fulminating type of typhus, or of epidemic meningitis. There is wild, aggressive delirium — a panphobia — the patient jumps out of the window, beats the plaster from the walls, eats bedding, and clutches at his attendant with the frenzy of despair; may sing, whistle, yell, and tear off clothing continuously for days. One patient kept plunging his head against the ceiling until beaten to a jelly. Another rubbed his thumb against his teeth until it hung by a thread.

Dr. Spitzka concludes, since "all the pathological changes of the brain noted post-mortem are only collateral results of disturbed circulation, and throw no direct light on the essential pathology of 'grave delirium,'" that we must infer the "formation of a toxic agent in the nerve-centres themselves."

Case I. ——, aged forty-two, American, married but deserted by husband; general health said to have been poor during her married life; had been "a little unlike herself" for the preceding five or six months. One week before admission to the hospital became actively insane, with extreme restlessness and increasing bodily depression. On admission showed most intense motor excitement which was most incessant for one week. This was followed by a week of great physical prostration in bed, ending in death on the fourteenth day, three weeks from the beginning of the attack. At times, during the last week, mind was quite clear. A few days before death a double pneumonia developed, which hastened the fatal issue. Urine contained albumin and hyaline casts on admission. Autopsy showed the large mottled kidneys of chronic, diffuse nephritis. Heart flabby, hypertrophied, and dilated. Liver enlarged. Lungs, red hepatization, except at apices.

Case II. ——, aged thirty-two, American, married, mother of three healthy children; paternal uncle insane; showed slight peculiarities for preceding year; apt to complain of "pain across back," but was not considered sick until ten days before admission, when violent insanity developed suddenly during the night; was kept in bed by mechanical measures, and was brought to the hospital, bound to a stretcher, over a distance of sixty miles. On admission she was in a semi-comatose condition; action of heart extremely feeble; urine loaded with granular and waxy casts and blood disks. She lived six days in this condition. Autopsy: Heart, organized clots in all cavities; aortic insufficiency. Kidneys, normal size, cortex thin and very fatty; one hemorrhagic infarct size of marble. Liver, "nutmeg" on section.

Case III. ——, aged twenty-nine, single, of Irish parentage, good family history. Reported good health up to three weeks before admission, when she became melancholy, with the delusion that the neighbors were defaming her character; persistently suicidal. On admission urine contained albumin, hyaline and granular casts; action of heart feeble. Death occurred after thirty-three days (in eighth week of attack), during which she was the subject of intense motor excitement with agonizing apprehensions. Once repeated rapidly for hours, in most piercing screams, "O God! where is my brother?" Fed with tube. No autopsy.

Case IV. ——, aged thirty-nine, of German extraction, wife of intemperate saloon-keeper, patient said to have drank beer in moderation; mother of eight children, three miscarriages during the preceding year said to have been artificially induced; domestic relations unhappy and general health not good for a year. Insanity developed suddenly, three weeks before admission, in the form of melancholia with suicidal and homicidal impulses; asked her friends "to put her in the asylum because she could not trust herself." On admission appeared well and continued sensible, cheerful, and industrious for three weeks; gave a clear history of her attack, and soon evinced a natural desire to go home and take her place in her family. One examination of the urine at this time gave negative results. Heart enlarged, with mitral systolic murmur. On twenty-second day she became melancholic; quiet, with tendency to stupor, but made several attempts to choke an unoffending fellow-patient; increasing mental dullness with rapid physical wasting for one week. On the eighth and ninth days (of this attack) showed extreme restlessness; would throw herself about the floor, beat the walls, scream, etc.; tenth day in bed; died on morning of eleventh day, A second examination of the urine made during this attack also gave negative results. Autopsy: Venous congestion everywhere marked; heart hypertrophied, with mitral leak; kidneys large, capsule thickened and removed with difficulty from a roughly granular surface; cortex, on section, showed extensive fatty change; liver also showed fatty changes in a few places.

This case is interesting as showing the remission which is so frequently seen in the mental, as in other manifestations of "Bright's disease," and also as illustrating the necessity for repeated examinations of the urine.

Case V. ——, aged thirty-three, Irish, single, domestic, good family and personal history. One week before admission suddenly became wildly delirious; no previous symptoms noted. On admission she was excited, incoherent, and in constant motion of some sort. Urine loaded with granular and waxy casts. Lived nine days; delirium passed into semi-comatose condition for the last two days of life. Autopsy: Kidneys contracted, capsule adherent, cortex thin, cystic, and fatty. Some thickening of heart valves and numerous atheromatous patches in coats of arteries of the brain.

Case VI. ——, aged twenty-six, American, single; one paternal cousin insane; general health had been considered good, but slight failure of memory and a tendency to repeat words had been noticed for two years. Onset of insanity sudden, twelve days before admission, shown by incessant talking of delusions of a persecutory nature, insomnia, and restlessness. On admission, condition as described above; after one week, passed into a condition of semi-coma, which lasted a month, and she died in the seventh week of the attack. The urine contained a small amount of albumin, hyaline, epithelial, and granular casts. There was a mitral systolic murmur of the heart. Ten days before death the urine contained a considerable amount of sugar, although none had been found at two previous examinations. In the last week showed a tendency to convulsive twitchings of all the voluntary muscles; superficial sores readily followed slight irritations of the skin. Autopsy showed contracted and granular kidneys. Heart hypertrophied, with mitral leak.

Case VII. ——, aged thirty-eight, married, mother of five children. Invasion of insanity sudden, of one week's duration; lived eight days, after admission to the hospital, in a condition of restless delirium with rapid loss of flesh. Autopsy showed kidneys contracted and fatty; heart hypertrophied and dilated.

Case VIII. ——, aged forty-five, of Irish parentage. Dyspepsia for two years, somewhat depressed for four months; melancholia, with restlessness and delusions, for one week before admission to the hospital. Special delusions were that there was "a roaring lion inside" her body; at another time a "man," etc. Continuously restless; died eighteen days from the beginning of the attack. No autopsy. Urine contained albumin; no casts found.

Case IX. ——, aged twenty-six, Pennsylvania German, single, domestic; left service six weeks before through ill-health, but there were no mental symptoms until two weeks before admission to the hospital, when she suddenly became deranged, screaming "The people are burning up!" Refused food and lost flesh rapidly. Died six days after admission, three weeks from the onset of mental symptoms. The urine contained albumin and many granular casts. The autopsy showed the kidneys much contracted, cortex granular and cystic.

CASE X. ——, aged thirty, American, married, good family history. Had suffered from dyspepsia, but was considered fairly well up to eight weeks before admission to the hospital, Insanity characterized by apprehensions of disaster, suicidal attempts, and great restlessness. Lived twenty-five days. No autopsy. Urine contained hyaline and granular casts.

CASE XI. ——, aged thirty-seven, American, widow, mother of five children. Had been running down in health for four months with some mental depression, but no obvious insanity until ten days before death, which was probably hastened by the fatigue of the journey to the hospital. She died after two days, during which she manifested great restlessness and mental distress, with apprehensions of injury. The urine contained albumin and granular casts. The autopsy showed kidneys granular and contracted, with a large cicatrix on the posterior surface of the left; heart very pale, flabby; insufficient valves.

CASE XII. ——, aged forty-seven, American, widow. Sudden development of insanity; melancholia, with great restlessness, fear, screaming, etc., followed by death in twenty days. Urine contained granular casts. No autopsy. This case was complicated by swelling of both parotid glands a few days before death.

*Second group.*—Cases (12) less rapidly fatal (three months to nine and one-half years).

CASE XIII. ——, aged fifty-nine, married, mother of two children; one maternal cousin insane. Melancholy for about two months before admission, with a remission of about one month, during which she seemed well; prominent mental features were delusions concerning her own body, apprehensions of injury and hallucinations of sight; at first very restless, but soon became quiet and markedly resistive; never stuporous, but seldom spoke; expression watchful, suspicious, and despairing: often required mechanical feeding; at times seemed to suffer great pain, which, on two occasions, appeared to be relieved by the passing of a large quantity of bloody urine. Emaciation was rapid and extreme. The urine contained albumin, hyaline, and epithelial casts. Death occurred in the seventh week of her residence with us, the fifteenth from the beginning of insanity.

CASE XIV. ——, aged thirty-two, American, married, mother of one child eight years old. Melancholy for nine months before admission; died six months later, fifteen from the beginning of insanity. No autopsy.

This case was characterized by extreme melancholy and strong suicidal impulses always active; generally silent with most agonized, despairing expression, but had periods when she would utter piercing screams and throw herself about the floor for hours; often fed with tube because she refused food. There were several slight convulsive seizures just before death.

CASE XV. ——, aged fifty, American, widow; no family history of insanity. Something more than a year before admission to the hospital became the subject of restless melancholia, followed by a gradual failure of intellect. On admission, bewildered and frightened air; comprehension feeble; spoke few words in disjointed, childish manner; appetite inordinate; habits unclean; destructive of clothing and other property; resistive to a marked degree, the most ordinary measures for her comfort requiring four to six nurses. Urine contained albumin (small amount), hyaline and fatty casts. This patient lived a year in the condition described above, more than two years from the beginning of the attack. No autopsy.

CASE XVI. ——, aged fifty, Irish, single; had been complaining of minor ailments for two years. Six weeks before admission became suddenly melancholy, with active delusions of persecution; suicidal; refused food and lost flesh rapidly. Died in the eighth month. Became very much emaciated; frequent abscesses followed superficial injuries; during a remission of three months was almost well, quiet, and sensible, but with this exception, was always restless, disorderly, and an exceedingly troublesome patient to care for. Urine contained albumin; no casts noted. Autopsy: Kidneys contracted, capsules adherent, cortex very thin and containing some cysts; pelves dilated and injected; heart valves thickened and atheromatous; atheroma of cerebral vessels.

CASE XVII. ——, aged forty, Irish, single, domestic; mother insane at menopause; second attack (first attack due to "ill-health," eighteen years before). One year before admission awoke suddenly in the night with the delusion that her room-mate was going to kill her. Lived seven months (nineteen from the beginning), always in a condition of exaggerated fear and apprehension. Gradual physical deterioration with development of tuberculosis. No record of urine. Autopsy: Kidneys contracted, cortex very much thinned; fatty degeneration of heart, with thickening of valves; lungs tuberculous, with almost total destruction of the right; two pints of purulent fluid in the right pleural sac.

CASE XVIII. ——, aged thirty-seven, American, married, and mother of five children; patient said to have been a chronic sufferer from "indigestion, liver and kidney troubles." Mental symptoms of four months' duration; a gradual development of melancholia with tendency to stupor. On admission, urine contained hyaline casts with granular epithelium. Mind and body failed together, and she died in four and one-half months, eight and one-half months from the beginning. Pulmonary tuberculosis developed in the later stages. Autopsy: Granular, contracted kidneys; tuberculosis of lungs; mitral stenosis of heart.

CASE XIX. ——, aged past fifty, married, no children, Irish; a niece insane. Had been considered fairly well up to about two months before admission, when she began to worry about trifles and developed hallucinations of sight and hearing; attempted suicide by cutting abdomen with a razor because she was "tired of herself." On admission, the urine contained hyaline casts; tendency to obesity, with flabby flesh and sluggish circulation; heart-sounds feeble. This patient seemed more hypochondriacal than truly melancholy; her attention could generally be diverted and at times she was even cheerful. Her suicidal intentions were not much credited until, one month after her admission to the hospital, she managed to secrete a table-knife and cut her own throat almost "from ear to ear," half severing the trachea, but no important vessels. This was followed by an amelioration of the mental symptoms. The wound kept in healthy condition and had healed to within one and a half inches (small opening into the trachea remained) at the time of her death, which was two months from the time of her admission, four from the beginning of her insanity. She was constantly threatened with heart-failure, and her death, occurring very suddenly, was evidently due to that cause. Autopsy: Kidneys a little swollen in appearance; capsule not adherent; cortex thin, friable, and of yellowish color; containing some cysts; pelves dilated and injected; heart flabby and fatty, valves thickened and atheromatous. Liver large and fatty; right lung contained, in posterior part of lower lobe, a gangrenous area, two by three inches, circumscribed by inflammatory adhesions of the adjacent pleura. Brain not examined.

CASE XX. ——, aged seventy-two, American, married; two brothers committed suicide. Suicidal melancholia of six months' duration; had a similar attack three years before, from which she apparently recovered. Lived two months, eight from beginning of last attack. No autopsy. Urine contained waxy and granular casts.

CASE XXI. ——, aged twenty-four, American, single, domestic; was "running down" in health for an indefinite time. Melancholia of restless type; rapid decline with development of tuberculosis. Urine contained a small amount of albumin. Died in four months. Autopsy: Granular, contracted kidneys; tuberculosis of lungs; mitral stenosis of heart; fatty liver.

CASE XXII. ——, aged sixty-six, American, widow. Melancholy for one year before admission, and committed suicide by hanging herself two months later. Urine contained albumin and hyaline casts. There was a mitral systolic murmur of the heart. No autopsy.

CASE XXIII. ——, aged sixty, Pennsylvania German, widow. Whole duration, nine and one-half years. Restless melancholia with keenest apprehensions constantly present. In the early stages would look out of window and scream "The sky is coming down!" "We shall be burned up!" in an agony of fear. Terminated in partial dementia of melancholic type, with extreme emaciation. Autopsy: Kidneys, cortex almost entirely destroyed by fatty change; heart, atheroma and thickening of valves; calculus in gall-bladder.

CASE XXIV. ——, aged sixty-five, American, widow, one daughter whose mental capacity seemed below the average. Two weeks before admission arose in the night and tried to set fire to the house. Condition that of restless melancholia of rather quiet type. Lived six and one-half years; without remissions; tendency to dementia slight. Autopsy: Kidneys contracted with extensive destruction of cortex; heart valves thickened; lungs tuberculous.

*Third group.*—Cases (8) terminating in rapid recovery.

CASE XXV. ——, aged thirty-seven, mulatto, married; ten days before expiration of a year's sentence in prison became melancholy, with keen apprehension of personal injury; hallucinations of hearing and of smell; refused food, "having heard them say that it was poisoned to kill her." On admission, urine contained a considerable amount of uric acid and a small amount of albumin; no casts noted. The mental syptoms disappeared in a few days and coincidently the urine became normal. She was discharged quite well in one month.

CASE XXVI. ——, aged forty-four, American, married, mother of seven healthy children. Had suffered from uterine trouble for seven years; internal hemorrhoids also. Simple melancholia developed two months before admission; lost interest in household and family; apprehensions of injury; headache, insomnia, "noises in head," etc. Urine contained hyaline casts and granular epithelium; heart normal. Recovered in six weeks (now two and one-half years ago).

CASE XXVII. ——, aged forty-nine, American, single, no insanity in family. Patient lived on a farm, in comfortable circumstances, and was accustomed to eating largely of food that was difficult of digestion; a large woman of full habit; melancholia developed very suddenly; distrusted her own family and jumped from an upper window with the idea of escaping impending danger. On admission (three days after beginning of insanity), mind much confused; incoherent, apprehensive, and especially resistive for about one week, after which convalescence was gradually established; discharged well in twenty-one days. (No relapse for three years.) Urine, on the third day, contained a large amount of uric acid and a few casts; on the eighteenth day it was normal.

Case XXVIII. ——, aged fifty-three, American, wife of a well-to-do farmer; two healthy children; grandfather and father committed suicide; sister and paternal cousin also have been insane. Patient was never strong; subject to palpitation of heart. At seventeen years had an attack of melancholia lasting "a few weeks." Four years ago was again melancholy for ten weeks, ascribed to "general debility." Present attack of three weeks' duration (irritable in her family for a longer period), restless, dissatisfied, and suspicious; suicidal tendencies suspected; physical condition poor; complained of "pains all over." On admission, found weak and anemic; the urine contained much uric acid, a considerable amount of albumin, and a few granular casts. There was a mitral systolic heart murmur. With improvement in her physical condition the mental symptoms disappeared, and she went home in three months quite well in mind; at that time examination of the urine showed a small amount of uric acid, but no albumin or casts. This patient has now been at home seventeen months; while I do not believe it possible for her ever to be well, in a physical sense, immunity from mental symptoms will depend upon the care with which the conditions of her life are regulated.

Case XXIX. ——, aged seventy-one, English, widow with two healthy children; a small delicately built woman. Two previous attacks of melancholia nine years apart. Present attack of four months' duration, appearing to follow erysipelas. On admission restless, apprehensive, suicidal. The urine contained a considerable amount of albumin, with many hyaline, granular, and waxy casts, which gradually diminished, with coincident improvement in the mental symptoms, until she was discharged well in two months from the time of her admission. At that time the urine contained an occasional cast, but no albumin.

In this case, as in the preceding one, the prognosis must be conditional. I cannot doubt in this, as in many similar cases that have come under my care, that the mental manifestations have been coincident with, and closely related to, exacerbations in the course of a slow, interstitial nephritis extending over many years.

Case XXX. ——, aged thirty-five, Pennsylvania German, married; father once insane, but recovered. Insanity, with frenzied excitement, developed two weeks before she was brought to me; had been tied down to prevent her injuring herself; would try to beat her head against the walls and floor, to dig out her eyes, etc. After admission, great restlessness continued for several days; sometimes would utter the most piercing screams for hours at a time; there was general confusion of mind, which gradually disappeared, but depression of spirits, apprehensions, etc., with remissions, persisted for a longer time. She went home well in six weeks, eight from the beginning of the attack. The urine, on admission, contained albumin, hyaline and granular casts. (It is much to be regretted that there is no record of the urine at the time of her discharge.)

CASE XXXI. ——, aged fifty, American, married, with two healthy children. Rheumatism one year before admission to the hospital; mental depression for two weeks, restlessness, apprehensive delusions, suicidal tendencies, and rapid physical deterioration; complexion markedly sallow; urine contained uric acid and granular casts; heart sounds feeble with a mitral systolic murmur. Under tonic treatment her physical condition improved, the mental symptoms disappeared, and she went home well in two months.

CASE XXXII. ——, aged forty-eight, American, single; very marked family tendency to insanity, and patient herself rather below the average mentally. A large healthy built woman. Fifteen days before admission to the hospital began to show mental disturbance with remissions; on the thirteenth day awoke from sleep with piercing screams; the same day had severe pain in the region of the heart, with dyspnea, lasting twenty minutes; micturition was reported as abnormally frequent during the first week, and she had complained of "pain in the back." On admission, face turgid, eyes injected, skin hot to touch; markedly resistive with general confusion of mind. Venesection was followed by immediate relief of the condition described, and on the second day she resembled nothing so much as a good-natured baby who is just learning to talk. She slowly regained her normal condition, and was discharged practically well in about two months. (One year later I have heard that there has been a relapse.) The urine contained uric acid, albumin, and casts, which diminished, but did not (the uric acid excepted) entirely disappear with her restoration to mental health.

*Fourth group.*—Cases (3) recovering after many months.

CASE XXXIII. ——, aged twenty-eight, American, wife of traveling salesman, with two healthy children; of healthy family. For the preceding year had complained of a "heavy pressure across the middle of the abdomen;" mentally deranged for seven weeks before admission, during which she was at first restless, with keen apprehensions of injury, followed by quiet with tendency to stupor. In the hospital was a typical case of "melancholia with stupor," showing little change for eight months, after which she gradually recovered. She went home in the tenth month; at that time her mind was slightly dull and worked slowly, but she has since been reported as entirely well (now nearly three years). The urine contained albumin, blood corpuscles, hyaline, epithelial and granular casts, during the first month, diminishing during the second, and disappeariug entirely after the third month.

Case XXXV. ——, aged thirty-three, Irish, single, domestic; in America over three years. For two years had been "running down" in health, with no very pronounced symptoms. Melancholy for four weeks before coming to the hospital; often cried loudly, saying "My soul is lost!" "Judgment day is coming!" Had in the time two short periods (three hours) of immobility. Had refused food almost entirely for two weeks. On admission, condition markedly resistive, and continued for about a month a mixture of stupor and obstinacy; often required mechanical feeding; a few times made violent attacks upon some one near, without warning or provocation; at the end of that time passed into a condition wholly passive and stupid; stationary for a year, then began to take some interest in the work of the ward, and gradually recovered from that time. The urine, on admission, contained a considerable amount of albumin, hyaline and granular casts; on discharge, no albumin, but a few hyaline casts. While practically well at the time of her discharge, it is doubtful if her mind had quite its former acuteness, and I look upon her as liable to a relapse.

Case XXXV. ——, aged twenty-five, single, parents Irish and German, worked in a mill; no insanity in family. Two months before admission came home from work sick, "seemed to have taken cold;" nine days later began to have melancholic delusions with increasing restlessness. With us she was tormented with self-condemnatory delusions for several weeks, with constant restlessness and suicidal impulses; then improved slowly, and after six months spent a few weeks at home, only to come back worse than before. At home had attempted suicide by setting fire to her clothing. Her mental anguish was now indescribable; for weeks she would walk the floor, wringing her hands, crying aloud and accusing herself; she was always in motion, picking at or tearing her clothing, scraping plaster from the walls, etc., if not constantly watched, not from mischievous propensities, but because she "could not keep still." This was two years ago. Six months ago she went home, and has engaged in business as a dressmaker. For a year before she was almost well, but seemed afraid to go home. The early records of the urine in this case I cannot rely upon; a year ago a few granular casts and a trace of albumin were found. Early in the case there were several attacks of apparent heart failure, and at her best she was subject to pain about the heart for days at a time; at times she had also pain in the left kidney. Pallor was always a noticeable feature; there was a mitral systolic murmur of the heart with accentuated second sound.

*Fifth Group.* — Cases (4) improved and nearly stationary for years.

Case XXXVI. ——, aged fifty-two, American, single, teacher, good family history. Insane fourteen years before she came under my care ; for the first two years of that time described as being "in a condition of great excitement," during which she made two attempts at suicide ; improved and lived at home some years with mind somewhat weakened. A few months before admission became more restless ; would leave her home and walk long distances with the idea of getting a position ; untidy in personal habits. On admission, December, 1884, walked up and down crying ; fretful, dissatisfied expression ; attention fixed with difficulty ; complexion very sallow. For the past four years has been much improved ; has gained flesh, reads and otherwise employs herself ; has the freedom of the grounds ; expression of face anxious and dissatisfied at times ; capacity for continuous application impaired. The urine contains a varying amount of albumin with granular casts. There is a mitral systolic heart murmur.

Case XXXVII. ——, aged forty-two, American, widow, with two healthy children. History of "pain in back" and headache for three years before admission, during which a natural disposition to worry had been greatly exaggerated, passing at length into delusions of persecution with apprehensions of injury. For about a year after she came to us she continued much depressed, but for the past six years has been much improved ; is rather "difficult" to get along with, and is apt to mistrust and misjudge others, but is able to live at home and works industriously. The urine, a year ago, contained a large amount of albumin and granular casts.

Case XXXVIII. ——, aged sixty, Irish, widow ; a former attack three years before ; insane one week before admission. On admission restless to extreme degree ; would throw herself about the floor and scream for hours at a time ; fed with tube for weeks. Improved after six months, and for the past two and a half years has been almost stationary ; memory and other mental faculties practically unimpaired, but she is inclined to worry without cause, and her temper is irritable and uneven ; insomnia is a marked feature. On admission the urine contained albumin, hyaline and granular casts ; at the present time (May, 1889,) considerable granular debris, but no albumin or casts.

Case XXXIX. ——, aged forty-five, German, wife of a tailor, with two healthy children ; no insanity in family. Eight months before admission had malaria, and about the same time became melancholy with self-condemnatory delusions and strong suicidal impulses. This patient was for many months one of our most miserably restless cases of melancholia and one of the most persistently suicidal cases I have ever seen. In the earlier stages she often made fierce attacks upon others, with the idea of being hanged if she killed some one. For about past three years now she has been quiet, helpful, and extremely kind to all about her, but she is always under observation as a suicidal patient, because, at times, the impulse comes back to her with overmastering strength. I have no reliable record of the urine at the time of her admission ; at the present time (1889) it contains albumin and casts.

*Sixth Group.* — Cases (3) running a very slow downward course.

CASE XL. ——, aged thirty-one, widow, American, of good family but married below her station and lived an irregular life for years ; became implicated, with her husband, in some breach of law, and was sent to prison, where melancholia developed, said to be a second attack. On admission, (October, 1887,) expression of face that of abject despair ; delusions self-condemnatory ; "God has cursed me!" the only words she spoke. Intensely suicidal ; at times homicidal (with the idea of subjecting herself to the death penalty if she could kill another). In the following Summer she was, for about a month, in a condition of semi-stupor, with restlessness and delusions that she was grossly maltreated, but for the remainder of the time she has been in the condition of despairing melancholy described above, with strong suicidal propensities always present ; she is subject to pain in the lumbar region and insomnia is a prominent feature of her case. The urine in this case has always contained casts, hyaline, granular, and sometimes waxy, with albumin in varying amount.

CASE XLI. ——, aged sixty-five, Irish, single, now in hospital nearly three years. Had been generally healthy, but had an "attack of diarrhea" three months before coming to the hospital, which left her weak ; two months later began to lose interest in her work ; wanted to wander away from home and mistrusted her friends ; finally did not eat, sleep, or change her clothing. On admission, she was in a restless, almost frenzied, condition, resisting everything ; in a month passed into a passive condition, in which she has remained, almost without change, to the present time. She is kept in bed, eats well when food is taken to her, submits passively to necessary care, but does not show any interest in anything about her, although her eyes are open and not devoid of intelligence. On admission the urine contained a few casts ; one examination, in 1889, showed albumin and an extraordinary number of casts.

CASE XLII. ——, aged fifty-six, of German extraction, wife of mechanic, mother of seven children ; one paternal cousin insane. Health broken down by "liver trouble" for the previous two years, during the last six months of which she was insane, with delusions of persecution and apprehensions of injury ; suspicious of her best friends, and made an attack upon her husband just before coming to the hospital. At times had periods of greater restlessness, when she would make efforts to escape from her supposed enemies. In the hospital (now eighteen months) she has generally been quiet ; face invariably expresses suspicion and misery ; at times "voices tell her to hang herself ;" occasionally restless and makes efforts to escape; complexion extremely sallow; for the past year slowly losing flesh. On admission the urine contained albumin and waxy casts, confirmed by subsequent examinations.

*Seventh group.*—Cases (2) illustrating a transformation of melancholia into secondary paranoia with delusions of personal grandeur.

CASE XLIII. ——, aged forty-three, of healthy family, American, single. Admitted to the hospital January, 1887, in a condition of melancholia of four years' duration, for the last nineteen months of which she had been in a private hospital for the insane. For the first nine months her whole attitude was that of the most profound dejection; for days she would sit with bowed head, refusing to speak, often to eat; then there would be an interval of a few days, when she would hold up her head, knit, and answer in monosyllables, if spoken to, but never smile and shunning observation. After nine months, (five and one-half years, it will be remembered, from the beginning of her insanity,) there was a sudden change and she became as exalted and active as she had previously been depressed and quiet, and her condition has remained practically unchanged for three years. A stranger meeting her might think her merely over-vivacious, with an incessant activity that is almost fatiguing to witness. She is not over ready to speak of her delusions, but if led to it depends her fixed belief that she is "the Queen of Heaven and will never die." She is always usefully employed and very helpful to all about her; she seems never tired. On admission the urine contained albumin (microscopic record not satisfactory). At subsequent examinations there has been a varying amount of albumin with hyaline and granular casts.

CASE XLIV. ——, aged thirty-eight, American, single, of good family, and always lived in comfort; never very strong; indulged from childhood, and not considered mentally equal to the rest of her family. Admitted to the hospital in September, 1884. "A few years" before had "chills and fever," which left her with pain in the back, apparently increasing in severity. Mentally deranged, with apprehensions of injury and hallucinations for two months before coming to me, for the last three weeks of which she was sick in bed. After admission to the hospital, she was weak and more or less in bed for several weeks, during which she had often fits of loud screaming; often fed with tube; delusions that "men came into her room at night to kill her;" that her "bed was filled with worms," etc. Gradual development of delusions of personal grandeur, that she is "Queen of the United States," "head attendant of the ward," etc. Otherwise mental action fairly good, and she is noted for her cheerfulness and activity. Her condition has been pretty uniform for the past three years. I have no satisfactory record of the urine at the time of admission. One examination, a year ago, showed a large amount of albumin, with granular casts. At the present time there is a trace only of albumin, with many granular casts. There is a mitral systolic murmur of the heart ; complexion a pale sallow.

(I omit two cases marked by extraordinarily varied hallucinations, with delusions of persecution, and one of stuporous melancholia merging into dementia, distinguished in the early stages by cataleptoidal tendencies.)

*Eighth group.*—Cases (4) of puerperal origin.

CASE XLV. ——, aged thirty-eight, American, married, and mother of three children. With first child, fifteen years before, had eclampsia and was unconscious for a week. Second child still-born ; labor began with convulsions, which continued for four days. During the third pregnancy, five years ago, urine was found loaded with albumin, and convulsions seemed to be averted by a free venesection. Since that time her memory has appeared to be slightly impaired, and she has been subject to fits of depression of spirits. Temper uneven for six months ; melancholia, with delusions, one month. Urine loaded with albumin just previous to admission. (For the foregoing notes I am indebted to Dr. R. B. Ewing, of Chester county.) On admission, (April, 1889,) in condition of pronounced resistive melancholia ; face turgid, skin hot, tongue brown and dry ; refused food and drink and resisted everything with a frightened, staring expression. The condition described was at once relieved by a very free venesection, which reduced the case to one of simple melancholia, in which the mind worked slowly but in natural lines. There was notable improvement for three weeks, then she went downward physically, losing flesh for a few weeks, and finally almost stationary ; her mental state, that of simple depression, for three months, when I advised a change for her. From her home she has written me several letters during the past nine months, which indicate that she has almost entirely regained her mental tone. She writes that she has a cough, but at the last report she had gained twenty pounds in weight. The urine, except immediately after the bleeding, has, at every examination, large amounts of albumin and granular casts. Heart fairly normal.

CASE XLVI. ——, aged thirty-two, of Irish parentage, married. Five years ago the birth of her first child was followed by a transitory attack of simple melancholia. Present attack came on a few days after the birth of her second child. On admission, eighteen months ago, she was in a condition of wild excitement, which I at first put down as a mania, but which more and more assumed the nature of melancholic frenzy with remissions ; before the nature of the case was fully apprehended, she had nearly taken her own life, and she has remained persistently suicidal. She is a most difficult patient to deal with, being an unusual combination of aggressiveness with suicidal proclivities. On admission the urine contained albumin, of which the relative amount has increased, and there are also many granular casts. The heart-sounds have been feeble and irregular.

CASE XLVII. ——, aged twenty-five, American, married, three children. Five weeks after the birth of her youngest child, jumped out of a chamber window "to save her baby;" had previously been dejected and had complained of "ice-cold feeling" at the base of the brain and of a peculiar itching of the soles. Was stuporous, but not profoundly so, during her stay with us, and recovery was gradually accomplished in eight months. On admission the urine contained granular casts.

CASE XLVIII. ——, aged thirty-two, mulatto, of superior intelligence; mother and two maternal cousins insane. Two months after confinement with her fourth child became melancholy. On admission very restless and markedly resistive, soon passing into a semi-stuporous condition, from which she never recovered. Died in three and one-half years of phthisis pulmonalis. The first examination of the urine, made soon after her admission, showed a few casts; but four subsequent examinations, made at different times, gave negative results. Autopsy: Cortex of kidneys fatty, diminished, in places absent; scrapings under microscope showed casts and fat globules; heart fatty, hypertrophied, and dilated; lungs tuberculous.

*Ninth group.*—Cases (2, also puerperal,) complicated with chorea.

In regard to the possible relation between chorea and the presence of "uremic" poisons in the blood, it is *á priori* conceivable that a condition which so often produces convulsions might also cause those other irregular incoördinate movements known as chorea. I find suggestive mention in some of the books on nervous diseases. Ross[1] accepts "a causal relation between rheumatism and chorea," and quotes the report of Dr. Mowry, who found "a rheumatic history" in twenty-nine to thirty-two per cent. of his cases of chorea.

Chorea rather frequently follows scarlet fever, a fact which Ross says "may probably be explained by the frequency with which scarlet fever is followed by rheumatism."

Both Ross and H. C. Wood[2] notice that chorea sometimes occurs during pregnancy and that cardiac murmurs are a frequent accompaniment.

1. Diseases of the Nervous System, Philadelphia, 1885, p. 680.
2. Nervous Diseases and their Diagnosis, Philadelphia, 1887.

Bristowe[1] says "chorea presents remarkable relations with heart disease, rheumatism, and scarlet fever."

Case XLIX. ——, aged twenty-eight, English, married. During her first and only pregnancy, five years ago, began to have fits of mental depression, which have continued to the present time; the attacks appear to be periodic, coincident with the menstrual epoch; at such times says she feels a strong impulse to kill herself. General choreic movements, of moderate intensity, have been constantly present for about two years. There is no visible impairment of the mental faculties, and her general condition is fairly good. The urine contains granular casts and oil globules, There is a mitral systolic heart murmur.

Case L. ——, aged twenty-six, American, married, with two children; domestic relations unhappy. General choreic movements appeared two months after the birth of youngest child, now about two years ago; ten months later began to have delusions concerning her own person — at times, of great wealth, etc. Was at other hospitals for a year, and minute history not obtained. On admission, (October, 1889,) mind very dull; kept in bed; exaggerated choreic movements of all the voluntary muscles. The urine contained granular casts and oil globules; heart action feeble. At the present time, has improved a little physically, and shows rather more intelligence; choreic movements unchanged.

*Tenth group.* — Cases (3) complicated with epileptiform convulsions — the "epileptiform melancholia" of some authors.

Case LI. ——, aged thirty-seven, American, wife of intemperate and abusive husband; two children. History of melancholia five months before admission; in third month had three epileptiform spasms in one night, followed by slight impairment of mind and melancholic delusions; in the fifth month also had several similar convulsions, followed by transient mental excitement. This patient has now been in the hospital eight years, condition pretty uniform throughout; there is slight mental enfeeblement, with tendency to mental depression; no delusions; she works industriously and has the freedom of the grounds. Complexion sallow and waxy. Reports of the urine in the early stages are wanting; there are now (May, 1889,) casts and albumin in considerable amount.

Case LII. ——, aged eighteen, Irish, single; father once insane through injury and recovered. Patient worked in mill; at fourteen years of age had an attack of malaria, of which weakness was a prominent feature; two years ago had a "bilious attack" and was admitted to our hospital (being then sixteen years of age) with general confusion

1. Diseases of the Nervous System, London, 1888.

of mind, tending to dulness and obstinacy — occasionally restless, unclothing herself. Recovered in three months. (No satisfactory record of urine at that time.) Returned in two years; at second admission urine contained hyaline casts. Quiet, mind slightly dull, with delusions of persecution, not very active. Had one convulsion a few days after admission, lasting two or three minutes, and, later, another, described by the nurse as epileptiform, and of considerable severity. She went home in six months, apparently well. About a year later I heard that there was a relapse, but as she did not come under my care it was probably transient.

CASE LIII. ——, aged forty-four, American, married, no children. Subject to headaches and "pain in the back;" for three months before admission, suffered from insomnia, loss of appetite, increasing mental depression, with apprehension of injury and suicidal tendencies. On admission, December, 1889, skin very dark sallow; emaciated; apprehensive, worrying, but mind increasingly dull. One examination of the urine at this time gave negative results. In the sixth week following had an epileptiform convulsion, followed by increased physical prostration. A subsequent examination of the urine showed casts and a large amount of fat. Her condition to the present time (June, 1890,) has been that of progressive mental and physical decline, and marked by no special symptom.

To the preceding I want to add notes of some recent cases that seem to me of special interest. The first two of these will illustrate the sudden invasion of melancholia, often so disastrous.

CASE LIV. ——, aged fifty-five, American, widow, mother of two robust children; no insanity in family; father died of cancer, brother and sister of phthisis. Patient had pneumonia twice, the last attack two years before date, following which her health was less good; she had periods of insomnia, and was more than formerly inclined to fret and worry. Twenty-three days before I saw her she had come to Philadelphia from her home in New York on a visit; at that time was under no medical supervision, but seemed to be in a condition of premature decay. Twelve days before I saw her she asked for a hammer "to fix her trunk," and went up to her bedroom, where she was found a few minutes later violently pounding her head with the hammer; she had made a large open wound of the scalp in the top of her head, and had bruised her face and temples. On admission to the hospital, we found her in a condition of general debility. Heart action very weak, sometimes irregular, with mitral systolic murmur. The urine contained many fine granular casts and fat-globules. She was at times very restless; often had self-condemnatory delusions; sometimes believed herself "possessed of the devil;" complained much of pain and crawling sensations in the top of the head. She remained with us only two weeks, and died at her home a few weeks later, her downward course marked by no special feature.

CASE LV. ——, aged thirty-nine, of German parentage, married, but deserted by husband; two children, youngest six months old. Had been considered ordinarily healthy; subject to rather frequent headaches, mostly frontal, for some years. One week before I saw her, on a rainy day, she had been out, and came in quite agitated, saying "Some one had given her poisoned candy;" soon became much excited and said there "were men in the room after her with pistols!" and jumped out of the window (first floor) to save herself. She was brought back and quieted, but during the following night jumped out of a chamber window, under the delusion that some one "was after her to kill her," fortunately sustaining no more severe injury than a sprained ankle. She was taken to a hospital the following day, and came to us at the end of the week. On admission, anemic, expression of exaggerated apprehension and fear, delusions of persecution and self-condemnation; extraordinary hallucinations of hearing — as the discharge of loud guns near her head, etc. Urine contained granular casts. Improved very rapidly in body and mind, and in three weeks appeared to be well. I was considering the advisability of discharging her, when she relapsed into a condition of acute mania, which has continued now two months; her physical condition is fairly good, and I regard the prognosis as favorable.

CASE LVI. ——, aged twenty-five, American, single; mother of feeble intellect and one brother now insane. Patient had been overtaxed in body and mind, when she was attacked by the epidemic influenza in the beginning of the present year, with considerable catarrhal lung trouble; she had no proper care, and was harassed by her insane brother, then at home. Three days before admission to the hospital she became suddenly and violently insane, On admission her mental excitement was very great, and in the form of mania, but it soon became intermittent and took the form of persecutory delusions. The urine contained granular casts at that time; a little later, while passing through an attack of erysipelas of the face, there were large quantities of albumin, granular and blood casts and blood disks, which diminished coincidently with abatement of the mental symptoms. Under treatment she recovered rapidly and well. (Two months.) I made one examination of the urine about a month after recovery, and found considerable granular detr[illegible]tus, no albumin or casts.

CASE LVII. ——, aged thirty-nine, of Irish parentage, single; general health always considered good up to January, 1890, when she had the epidemic influenza, took no care of herself, has been in poor health since. For a month previous to admission, April 10, 1890, had delusions that her family wanted to poison her ; insomnia and restlessness prominent features ; tried to get away from home. On admission, urine contained albumin and granular casts. Mental condition as described ; expression despairing ; refused food and at times fed with tube. At present (June) is improving,

CASE LVIII. ——, aged forty, Pennsylvania German, single, domestic ; general health has been considered good ; not as well as usual for two weeks, but kept at wor kuntil three days before admission, (May 31, 1890,) when she jumped out of her chamber window to escape imagined dangers. On admission, mind confused, with tendency to stupor, for a few days ; then a short lucid interval, followed by a condition characterized by suspicion and obstinacy. The urine contains granular and epithelial casts. Her general condition is improving and she will probably recover.

CASE LIX. ——, aged thirty, American, married, mother of four children, of which the youngest is four and one-half months old. Subject to frequent "sick headaches" for years ; said to have heart trouble and suffers from hemorrhoids. About six weeks after the birth of her last child was taken with the epidemic influenza ; "severe pain running up the back and stopping in the top of the head," the most prominent feature of the attack, from which she never got up well. Melancholia, with delusions, showed itself almost immediately ; said that there was "an evil spirit following her and controlling her actions ;" accused herself of having "murdered" her child, etc.; insomnia, loss of appetite, periods of great restlessness and rapid physical prostration. Once escaped from her family during the night. General condition since admission (May 26, 1890,) very poor ; anemic. Urine contains granular casts and fatty epithelium ; heart weak, with loud mitral systolic murmur ; mentally as described above ; prognosis doubtful.

CASE LX. ——, aged thirty-two, American, married, mother of two children, youngest nine months old ; a paternal cousin is an epileptic. Had malaria in June, 1888, and has been runni ıg down in health ever since ; additionally depressed by a moderate attack of the influenza, in January of this year. Has a slight laceration of the cervix uteri, and was in the woman's hospital for six weeks, leaving there two weeks before admission to the Norristown Hospital. About that time began to awaken suddenly from sleep, in the night, in a sort of terror ; once tried to get out of the window in her fear. Every night would say: "This is my last night to live!" The urine contains granular casts and much granular epithelium ; there is a loud mitral systolic heart murmur ; complexion very sallow ; general condition poor. Now in hospital two weeks ; quiet, with self-condemnatory delusions. For the past two months said to have complained much of "creeping sensations in the back of the head and neck ; "pain in the back" has also been a common symptom for two years.

